# Liquid Biopsy and Artificial Intelligence as Tools to Detect Signatures of Colorectal Malignancies: A Modern Approach in Patient’s Stratification

**DOI:** 10.3389/fonc.2022.856575

**Published:** 2022-03-08

**Authors:** Octav Ginghina, Ariana Hudita, Marius Zamfir, Andrada Spanu, Mara Mardare, Irina Bondoc, Laura Buburuzan, Sergiu Emil Georgescu, Marieta Costache, Carolina Negrei, Cornelia Nitipir, Bianca Galateanu

**Affiliations:** ^1^ Department II, University of Medicine and Pharmacy “Carol Davila” Bucharest, Bucharest, Romania; ^2^ Department of Surgery, “Sf. Ioan” Clinical Emergency Hospital, Bucharest, Romania; ^3^ Department of Biochemistry and Molecular Biology, University of Bucharest, Bucharest, Romania; ^4^ OncoTeam Diagnostic S.A., Bucharest, Romania; ^5^ Department of Toxicology, University of Medicine and Pharmacy “Carol Davila” Bucharest, Bucharest, Romania; ^6^ Department of Oncology, Elias University Emergency Hospital, Bucharest, Romania

**Keywords:** liquid biopsy, colorectal cancer, patients stratification, artificial intelligence, robotic surgery

## Abstract

Colorectal cancer (CRC) is the second most frequently diagnosed type of cancer and a major worldwide public health concern. Despite the global efforts in the development of modern therapeutic strategies, CRC prognosis is strongly correlated with the stage of the disease at diagnosis. Early detection of CRC has a huge impact in decreasing mortality while pre-lesion detection significantly reduces the incidence of the pathology. Even though the management of CRC patients is based on robust diagnostic methods such as serum tumor markers analysis, colonoscopy, histopathological analysis of tumor tissue, and imaging methods (computer tomography or magnetic resonance), these strategies still have many limitations and do not fully satisfy clinical needs due to their lack of sensitivity and/or specificity. Therefore, improvements of the current practice would substantially impact the management of CRC patients. In this view, liquid biopsy is a promising approach that could help clinicians screen for disease, stratify patients to the best treatment, and monitor treatment response and resistance mechanisms in the tumor in a regular and minimally invasive manner. Liquid biopsies allow the detection and analysis of different tumor-derived circulating markers such as cell-free nucleic acids (cfNA), circulating tumor cells (CTCs), and extracellular vesicles (EVs) in the bloodstream. The major advantage of this approach is its ability to trace and monitor the molecular profile of the patient’s tumor and to predict personalized treatment in real-time. On the other hand, the prospective use of artificial intelligence (AI) in medicine holds great promise in oncology, for the diagnosis, treatment, and prognosis prediction of disease. AI has two main branches in the medical field: (i) a virtual branch that includes medical imaging, clinical assisted diagnosis, and treatment, as well as drug research, and (ii) a physical branch that includes surgical robots. This review summarizes findings relevant to liquid biopsy and AI in CRC for better management and stratification of CRC patients.

## 1 Introduction

Colorectal cancer (CRC) is the second most frequently diagnosed type of cancer and a major worldwide public health concern (1, 2). Despite the efforts in the development of modern therapeutic strategies, the prognosis of CRC mostly depends on the disease stage, and specific biological features. Despite that the management of CRC patients has been based for decades on robust diagnostic methods such as colonoscopy, histopathological analysis of tumor tissue, molecular biology assays for molecular profiling, imaging methods (computer tomography or magnetic resonance), and serum tumor markers analysis (Carcinoembryonic antigen (CEA), Carbohydrate antigen 19-9 (CA 19-9), etc.), these strategies still have many limitations and do not fully satisfy clinical needs due to their lack of sensitivity and/or specificity to early disease detection even before clinical onset. Clinical investigations that leverage knowledge on tumor classifications enabled the development of new treatments by providing output data from effective screening and surveillance strategies. Therefore, improvements of the current practice would substantially impact the management of CRC patients and this review provides an in-depth particular discussion on two major approaches that hold great promise in this respect. On one hand, liquid biopsy, as a minimally-invasive procedure that involves the real-time analysis of tumor-derived material isolated from different body fluids (3) is a promising tool that could improve the current practice by helping clinicians screen for disease, stratify patients to the best treatment and monitor treatment response and resistance mechanisms. On the other hand, the prospective use of artificial intelligence (AI) in medicine holds great promise in oncology, not only for early and precise diagnosis but also treatment, therapy, and prognosis prediction of disease.

## 2 Standardization in Colorectal Cancer Molecular Profiling

Significant advances have been made in the past 20 years with respect to diagnostic by refining tumor classification, enabling this way pathologists and molecular biologists to better define both pathologic and molecular colorectal cancer features (4). In the current clinical landscape, the most common tools used for CRC classification are the histological subtype, tumor localization, and the molecular pathway underlying carcinogenesis (5). Regarding the histopathology of CRC, adenocarcinoma is the most common type of CRC, accounting for more than 90% of the CRC cases diagnosed worldwide, including mucinous adenocarcinoma (10%) and signet ring cell CRC (2%) (6, 7). Other rare types of CRC are medullary, squamous cell, adenosquamous, micropapillary, serrated, cribriform comedo−type, spindle cell, neuroendocrine, and undifferentiated carcinomas (8–10), that are more aggressive and present a significantly worse prognosis than classic adenocarcinomas (11). More, due to the embryonic origin, local vascular and nervous anatomy as well as the distribution of the gut microbiota, the tumor localization along the colon can be divided into: right sided tumors arising from cecum, ascending colon, hepatic flexure and/or transverse colon up to splenic flexure and left sided tumors arising from descending, sigmoid and/or rectosigmoid parts of the colon, potentially influences progression, prognosis and therapy. As a result of the different physiological functions of the colon segments, the patient’s clinical symptoms also significantly differ: right sided colon cancer frequently determine occult blood loss and consequently, iron deficiency anemia, while left sided colon cancer indices changes in bowel habits (12, 13). Finally, a complex picture of the CRC can be obtained by identification of the molecular pathways underlying CRC carcinogenesis, at least three major molecular pathways being related to CRC development and progression: chromosomal instability pathway (CIN), microsatellite instability (MSI), and the CpG island methylation pathway (CIMP), pathways that are not mutually exclusive (14–16). Most frequently identified (>85% cases) is CIN, which presents as tumor genetic signature aberrations in oncogenes and/or tumor suppressor genes involved in key signaling pathways for cancer initiation and progression such as APC, KRAS, NRAS, PIK3CA, BRAF, and TP53 (17–20).

Considering the heterogeneity of tumors sharing the same histology, starting with 1963, the World Health Organization (WHO) implemented a worldwide standardization by classifying the tumor diagnosis and providing internationally acknowledged standards. Currently, in its fifth edition (21), this classification is organized considering the tumor site, category, family, and type and, in addition to histopathology, it includes clinical appearances, epidemiology, etiology and pathogenesis, imaging studies, genetics, epigenetics, and other molecular investigations knowledge, highlighting the expanding concern of the multidisciplinary approach. The WHO new classification of tumors includes for the first time a volume exclusively dedicated to the Digestive System Tumors that now contains separate chapters for soft tissue and hematolymphoid tumors, inherited genetic tumor susceptibility syndromes and a broad classification of neuroendocrine tumors and carcinomas, malignancies that share common histology but occur in different anatomic sites (22). This classification was possible due to the accelerated growth in understanding cancer biology that includes the unrevealing of the genomic makeup of all known cancer types. Currently, sequencing of tumor-derived DNA is used as an investigational tool, aiming to match patients with their most appropriate treatments based on their particular target mutations. US Food and Drug Administration (FDA) labeling, National Comprehensive Cancer Network (NCCN) guidelines, conference proceedings, disease-focused expert group recommendations, and the scientific literature are relevant sources of information regarding the clinical significance in a specific gene’s alteration. To ease the interpretation of the genomic alterations detected in patient tumors, several support tools such as My Cancer Genome (23), CIViC (24, 25), the Precision Medicine Knowledge Base (26, 27), The Jackson Laboratory Clinical Knowledgebase (28, 29), Cancer Genome Interpreter (30), Cancer Driver Log (31, 32), Tumor Portal (33, 34), Targeted Cancer Care (35), Personalized Cancer Therapy (36, 37), and OncoKB (38) were developed. For example, OncoKB is the first racking that assigned each mutation to one of four levels defined based on the data availability supporting the use of the mutation as a predictive biomarker. Off-label use of cancer drugs in oncology has been practiced for many years (39–41) especially in the case of rare cancers where a randomized clinical trial may not be done. Notably, there are particular situations where FDA-approved drugs are not warranted as off-label use due to clear evidence of low efficiency. Such an example is vemurafenib, a BRAF inhibitor and a standard treatment option for patients with BRAF^V600E^ mutant melanoma or NSCLC, which is not efficient at least as monotherapy in patients with BRAF^V600E^ mutant CRC (42). More, aiming to support oncologists to assign potential targets when a broad gene sequencing panel is available, the European Society for Medical Oncology (ESMO) proposed a six levels of evidence framework classifying targets for precision cancer medicine (43). In this sense, the ESMO Scale for Clinical Actionability of Molecular Targets (ESCAT) has been developed (44). It measures how targetable colorectal tumors’ gene alterations are, based on data that support the effectiveness of their corresponding drugs. By far, the most important in terms of treatment decision are hot-spot RAS mutations (NRAS and KRAS), which predict resistance to epidermal growth factor receptor (EGFR) therapy. Decisions in metastatic colorectal cancer in the first line are made depending on this and the location of the tumor (right colon versus left colon) (45–47). From a molecular point of view, right sided colon cancer and left sided colon cancer are very different as the first one is frequently associated with defective mismatch repair genes, KRAS and BRAF mutations and miRNA-31, while the latter is commonly associated with CIN, p53, NRAS, miRNA-146a, microRNA-147b, and microRNA-1288 (48). As a consequence, this different molecular makeover of the tumors impacts on the therapeutically approach (49–51). The BRAF^V600E^ mutation is next as importance for treatment decision. The prevalence of genetic alterations in CRC was reported in the literature as follows: KRAS mutation 44%, NRAS mutation 4%, BRAF mutation 8.5%, NTRK1 fusion 0.5%, ERBB2 amplification 2%, PIK3CA hotspot mtation 17%, ATM mutation 5%, MET amplification 1.7%, RET fusion 0.3%, ALK fusion 0.2% (44). The combination of encorafenib and cetuximab has been shown to be effective in terms of overall survival in patients with this mutation according to a phase III study (52). Another very useful information in the therapeutic decision is about microsatellite instability. Patients with altered mismatch repair proteins (MLH1, MSH2, MSH6 and PMS2) had a very good oncological response to pembrolizumab and nivolumab in phase 2 and 3 trials (53, 54). Other gene alterations that may influence the therapeutic decision are NTRK fusions (which may correspond to an NTRK inhibitor, like entrectinib in the metastatic stage) or the amplification of ERBB2 (which can be associated with dual anti-HER-2 blockade) (36, 55, 56).

## 3 Liquid Biopsy in Colorectal Malignancies

**Figure 1:**
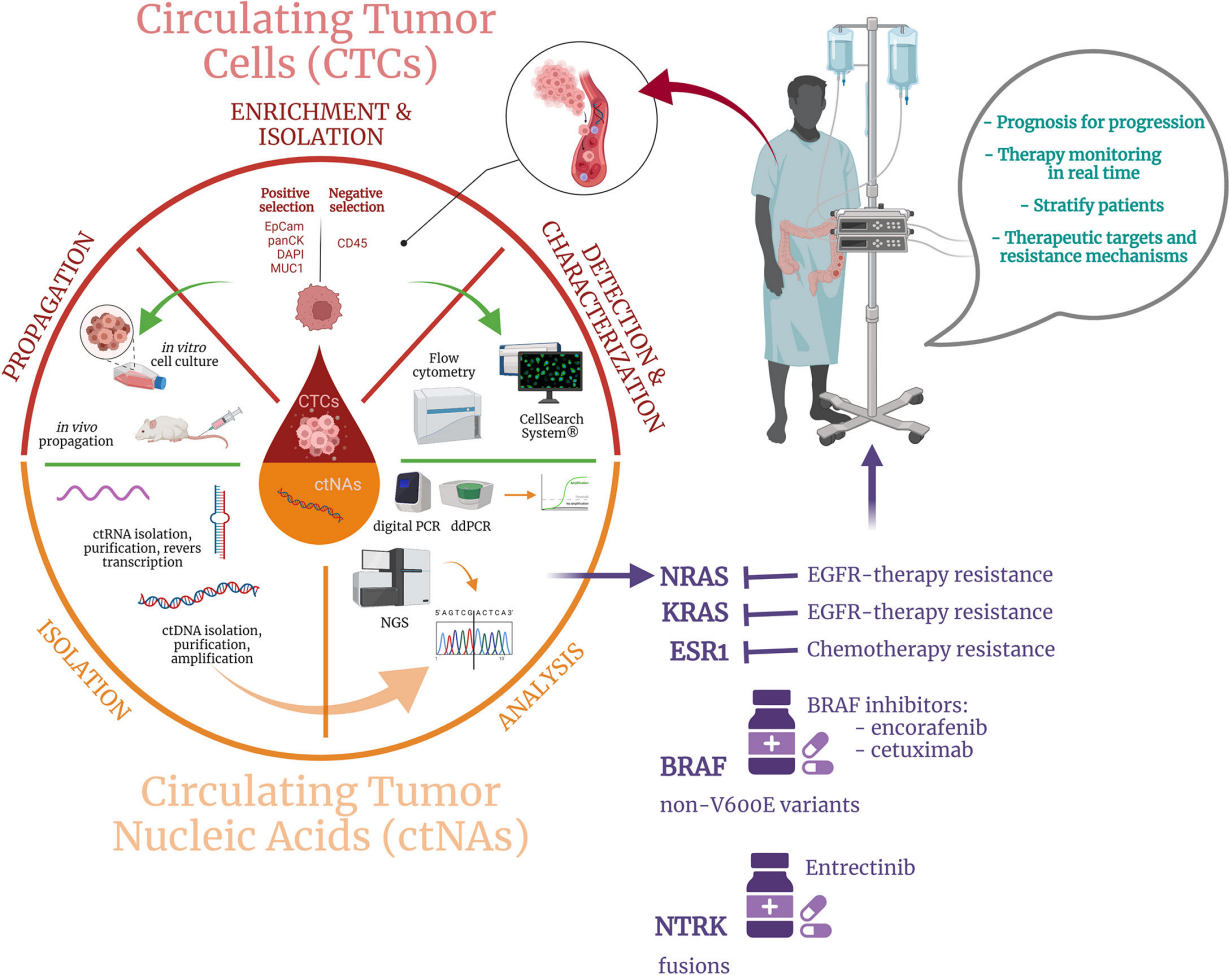
Schematic depiction of the liquid biopsy approach including: (i) CTCs and ctNAs enrichment, isolation, and characterization by specific techniques, (ii) output data analysis, and (iii) potential benefits for CRC patients.

For a long time, solid biopsy, a procedure based on analyzing tissue samples harvested during endoscopic tests or surgical specimens, was the only available option for diagnosis and tumor profiling in CRC patients’ management and it still represent the standard approach, widely available in clinical practice (57, 58). Tissue biopsy represents a static snapshot of the tumor that lacks to capture the CRC intratumor heterogeneity and dynamic evolution of CRC disease, also determined by clonal pressure induced by the administered treatment (59). More, it is an invasive procedure, which cannot be commonly repeated on-demand, making this approach unfeasible to be performed as a routine procedure for CRC patients’ long-term monitoring and treatment readjustment. As solid biopsy still represents a critical tool for CRC diagnosis, staging, and tumor molecular characterization at the sampling time, the disadvantages associated with this classical approach opened the opportunity for exploring novel analysis methods to improve the current clinical practice in CRC. In this context, the appearance of liquid biopsy represented a game-changer for the current clinical landscape and holds great promise for improving the ongoing CRC patients’ approach in terms of diagnosis, prognosis, and treatment personalization. Liquid biopsy is considered a surrogate of the traditional biopsy, being a minimally-invasive procedure that involves the real-time analysis of tumor-derived material isolated from different body fluids, such as peripheral blood, urine, pleural liquid, saliva, or ascites (3). Based on the anatomical localization of the CRC primary tumors and metastases, peripheral blood remains the main sample employed for liquid biopsy analysis, although urine can be also a viable alternative (60, 61). Unlike traditional biopsy, this modern approach is not limited spatially and temporally and provides clinical decision-worthy information associated with both primary and metastatic cancerous lesions. Among tumor-derived components, circulating tumor cells (CTCs), cell-free circulating nucleic acids (cfNAs), and extracellular vesicles (cEVs) are the most extensively studied and well-characterized markers in connection with CRC and are used for different purposes such as early cancer detection, staging, prognosis, or drug resistance and minimal residual disease (MRD) monitoring (**Figure 1**).

### 3.1 Circulating Tumor Cells (CTCs)

The first biomarkers associated with the liquid biopsy concept were the CTCs, but their discovery is not state of the art. Since 1869, when Thomas Ashworth first reported the presence of a subpopulation of cells in the bloodstream of metastatic cancer patients, cells that shared similar features to the primary tumor where they detached from (62), numerous successive studies reported the existence of CTCs and their role in the metastatic cascade (63–65). However, since the number of CTCs is extremely low, the promotion of these circulating biomarkers as promising tools in oncological clinical practice was dependent on the development of novel analysis methods with superior sensitivity that emerged from the recent progress made in the biomedical field (66). The frequency of CTCs found in circulation is directly related to the tumor burden, but even in metastatic CRC (mCRC) patients, only 1-10 CTCs/mL of blood can be detected in peripheral blood samples (67). Considered initiators of the metastatic cascade, CTCs are cells of epithelial origin that shed into the blood and lymphatic systems, exit routes that allow them to colonize distant tissues and generate secondary cancer lesions. These cells depart from solid tumors as single intact cells or cell clusters, a feature that impacts their metastatic potential and half-life in circulation (68). CTCs can agglomerate and form homotypic cell clusters or can interact with macrophages, platelets, and/or stromal cells to form heterotypic clusters (68). Of note, in mCRC, CTCs originate both from primary and metastatic lesions, showing the effectiveness of these biomarkers to capture the global landscape of CRC tumor heterogeneity (69, 70). However, independent of the departure point, the capacity of CTCs to cause metastatic lesions is dependent on their stability in circulation, as most of CTCs die as a result of their inability to surpass physical and oxidative stress, anoikis, and absence of the tumor-specific microenvironment (71–73). Based on studies performed on preclinical models, it is estimated that of all detached CTCs, 0.1% remain viable in circulation, from which only 0.01 – 0.2% ultimately will grow into a metastatic lesion (74).

CTCs are characterized by the expression of the epithelial cell adhesion molecule (EpCAM) (75) and the cytokeratins (panCK) (76). However, there are serious concerns on using EpCAM only to capture and define CTCs as these cells undergo epithelial to mesenchymal transition (EMT) and down-regulate the expression of this marker (77, 78). Additional markers MUC-1 (79, 80) could be also relevant for targeting CRC cells. More, circulating cells of tumor origin undergo epithelial to mesenchymal transition (EMT) a process that was first identified in embryogenesis and that refers to epithelial cells reprogramming by acquiring a mesenchymal phenotype. EMT is involved in crucial physiological processes such as development and wound healing, but it is also involved in malignant progression. Upon activation of this reprogramming process, CTCs undergo a series of physical changes that enable their long-distance dissemination, invasion, and survival in the bloodstream (81).

Due to the rarity of CTCs in circulation, the identification, capture, and analysis of these cells are challenging issues. In this view, to improve the success of CTCs detection, the process is preceded by an enrichment technique to unmask these low-frequency cells from the myriad of blood cells: white blood cells (5-10 × 10^6^/mL), red blood cells (5-9 × 10^9^/mL), and platelets (2.5-4 × 10^8^/mL) (67). The enrichment strategies for CTCs detection take advantage of their specific physical and biological characteristics in terms of size, density, deformability, or specific cell surface markers (82). Among all the enrichment strategies explored, the most popular choice remains immunocapturing which can be performed either for CTCs collection (positive enrichment) or removal of blood cell subpopulations (negative enrichment).

The CellSearch^®^ platform is so far the only system that has been approved by the Food and Drug Administration (FDA) for CTCs identification, isolation, and enumeration in whole-blood samples collected from metastatic colorectal (83), breast (84, 85), and prostate cancer (86) patients. The technology uses a positive enrichment strategy based on selection and immunomagnetic capture of Epithelial Cell Adhesion molecule (EpCam) positive cells (87) through ferrofluidic nanoparticles functionalized with the EpCam antigen that select and capture a subpopulation of cells which are subsequently discriminated from residual hematopoietic cells by CD45 negative expression after an immunofluorescence assay. Using the CellSearch^®^ system, EpCam positive cells that are cytokeratin^+^/DAPI^+^/CD45^-^ are selected as CTCs and counted for cancer prognosis (88, 89). The platform was successfully employed in various studies that aimed to quantify CTCs in CRC patients and revealed the importance of these as independent prognostic markers (66, 90, 91). However, despite being the only system FDA approved, this platform presents several disadvantages such as low sensitivity and specificity and most important, the failure of detecting CTCs that lose their epithelial features as a result of EMT (92, 93). As a result of undergoing EMT, CTCs acquire a mesenchymal phenotype and thus lack specific epithelial markers such as EpCam and cytokeratins (94), pillar molecules for numerous CTCs enrichment and detection strategies, including the FDA-approved CellSearch^®^ System. From this point of view, Flow Cytometry which is extensively used in hematology could be successfully employed in CTCs detection and count as a versatile approach considering that the instruments work with full customizable protocols. Fluorescence-activated Cell Sorting (FACS) which is also a flow cytometry assay allows also the isolation of the CTCs sorted based on their specific markers expression. Other methods for CTCs isolation, divided by the enrichment strategy, are presented in **Table 1**.

**Table 1:** Methods for CTCs isolation divided by the enrichement strategy.

Enrichment Strategy	Technology	Selection Criteria	Ref.
*Immunomagnetic enrichment*			
*Immunomagnetic positive enrichment*	CellSearch^®^	EpCam	(66)
	MagSweeper	EpCam	(95)
	Magnetic Activated Cell Sorter (MACS)	EpCam	(96)
	Strep-Tag	EpCam, EGFR, HER2	(97)
	Immuno-magnetosomes (IMS)	EpCam	(98)
	AdnaTest	EpCam	(99)
*Immunomagnetic negative enrichment*	EasySep	CD45	(100)
	RosetteSep	CD45	(101)
*Microfluidic positive capture*	CTC-Chip	EpCam	(102)
	Isoflux	EpCam	(103)
	Nanovelcro	EpCam	(104)
	High-throughput Microsampling Unit (HTMSU)	EpCam	(105)
	Verifast	EpCam	(106)
*Size-based enrichment*			
*Microfluidics*	Parasortix	Gap sizes from 10 μm down to 4.5 μm	(107)
	Microcavity Array (MCA)	8-μm circular cavities	(108)
*Density-based*	OncoQuick	Density	(109)
	AccuCyte	Density	(110)
*Membrane filtration*	Isolation by Size of Tumor cells (ISET)	8 μm pores	(111)
	Flexible micro spring array (FMSA)	8 μm pores	(112)
	ScreenCell	6.5 µm pores	(113)
	Fluid Assisted System Technology (FAST)	8 μm pores	(114)
*Dielectrophoresis*			
*Dielectric properties*	ApoStream	Polarizability	(115)
*In vivo*			
*Therapeutic apheresis*	Diagnostic leukapheresis	EpCam	(116)
	GILUPI CellCollector	EpCam	(117)

The low frequency of CTCs in blood severely impacts the clinical utility of these biomarkers for CRC screening and early detection as CTCs detection is almost impossible in early-stage cases (118). In this case, their detection has been a challenging issue that is reflected by the reduced number of studies available regarding the use of CTCs as CRC screening and early diagnosis tools. However, promising results for employing CTCs in screening protocols were shown by Yang et al. (119) that used a negative enrichment strategy for CTCs detection by fluorescence *in situ* hybridization in blood samples of patients with colorectal polyps and non-metastatic CRC. This study revealed the presence of CTCs in both benign and malign disease, with a significant increase of CTCs in the metastatic setup, where the CTCs count was modulated by the tumor anatomical location and degree of tissue differentiation. More, Tsai et al. employed the CellMax biomimetic platform to detect and enumerate CTCs in subjects with adenoma, polyps, or stage I-IV CRC and revealed an 88% accuracy of this approach for detection of CTCs in all tumor stages, including precancerous lesions (120). The CellMax platform’s unique design enables a superior detection method of CTCs in terms of sensitivity and purity and leads to recovery of viable CTCs that can be employed in downstream applications, making this approach appealing not only for early detection of CRC (121). Another approach to overcome the low frequency of CTCs for early CRC cancer could be repositioning diagnosis leukapheresis (DLA) as a CTCs enrichment tool based on the similar density these cells have with the mononuclear cells from peripheral blood (116, 122, 123), a method that has been successfully employed by Soya and colleagues for CRC patients (124).

Nevertheless, the most common clinical utility of CTCs remains cancer prognosis, a high count of CTCs being associated with a worse prognosis. A prospective study on preoperative patients with stage I-IV CRC showed that CTCs frequency, quantified using the CellSearch^®^ system, was increased in mCRC patients as compared with non-mCRC patients. For non-mCRC, CTCs detection represented a strong and independent prognostic marker and was associated with worse overall survival (OS) and progression-free survival (PFS) compared to CTCs negative patients (90). Using CTC for mCRC patients exclusively revealed that the quantification of CTCs represents an independent predictor of PFS and OS as both indicators were shorter for patients with ≥3 CTCs/7.5mL as compared with those of patients with <3 CTCs (83).

Regarding the clinical implications of CTCs analysis with impact on the post-operative therapy regimen setting in early and mCRC, Guadagni et al. (125) showed that CTCs from 79% of patient exhibited moderate to high sensitivity to mitomycin as compared to only 7% for irinotecan, 30% for oxaliplatin, 2.3% for 5-fuorouracil, 14% for raltitrexed, 4.6% for alkeran, and 4.6% for carboplatin. More, the same group used CTCs to select drug regimens and subsequent target therapy that was subsequent successfully administered by hepatic artery infusion to patients with colorectal cancer liver metastasis (126).

### 3.2 Circulating Tumor Nucleic Acids (ctNAs)

The limited clinical applicability of CTCs for CRC diagnosis and treatment personalization has led to a shift of liquid biopsy studies in recent years to employ preferentially cell-free circulating nucleic acids (cfNAs) in CRC patient management, cfNAs including cell-free circulating DNA (cfDNA) and cell-free circulating RNA (cfRNA). Indeed, analyzing cfNAs by molecular technologies can lead to real-time genomic profiling of the tumor, an extremely useful approach for the modern trends in CRC management that are directed towards precision medicine and patient-oriented treatment. As the tumor molecular profile dynamically changes in response to the immune system and administered drugs, it is mandatory to implement a novel analysis tool that can be used on demand for ensuring the appropriate treatment regimens for CRC patients (127).

cfDNA is comprised of multiple degraded fragments of DNA that are released in circulation by cell apoptosis, necrosis, or active release mechanisms (128, 129). Apoptosis generates short fragments of cfDNA (< 1000 bp) while following necrosis longer fragments are released (130). The presence of cfDNA in circulation is not exclusively associated with cancer, and this observation was made since its discovery in the blood plasma harvested from healthy subjects (131). However, in cancer patients, the levels of cfDNA are increased due to the supplementary cfDNA released by tumor cells. This small fraction (<1%) of cfDNA, known as cell-free circulating tumor DNA (ctDNA), originates from primary tumors, metastases, or CTCs and harbors specific tumor-related alterations that mirror the genomic status of CRC tumors and epigenetic alterations, thus unraveling the tumor molecular make-up (130, 132). More, cfDNA levels are directly dependent on the cancer stage, mCRC patients showing significantly increased levels of cfDNA in comparison with non-mCRC patients, highlighting the prospective use of cfDNA analysis for early detection of CRC (132–134). More, the ctDNA concentrations are correlated not only with CRC stage, but also with tumor size (135), and could be also employed as a prognostic tool since high levels of cfDNA reveal a shorter OS (136).

In early CRC, ctDNA can be used for investigating DNA methylation, a feature that is closely related to initiating and sustaining the transition of colon polyps to CRC (137). This alteration can be monitored through the FDA-approved Epi proColon^®^ assay, designed for identifying septin-9 (SEPT9) methylation in CRC patients. SEPT9 gene is a tumor suppressor gene that loses its role as a result of hypermethylation of the CpG island in the promoter region, a molecular aberration that promotes the development of CRC (138). The Epi proColon^®^ test aids the early detection of CRC and showed good sensitivity and specificity when compared with the other available non-invasive tests: stool-based guaiac fecal occult blood tests (FOBT) and fecal immunochemical tests (FIT) and blood-based CEA test (139, 140). A study conducted by Tóth and colleagues (141) that included both left- and right-sided CRC cases revealed that this assay is a reliable screening method in both cases, showing superior sensitivity and specificity than FOBT and CEA. Superior performance was observed also comparing SEPT9 assay with FIT in terms of sensitivity (73.3% vs. 68%), while the specificity was significantly better for FIT (97.4% vs. 81.5%). However, the combinatorically approach of using both tests improved these numbers to 88.7% sensitivity and 78.8% specificity (142).

At the moment, for ctDNA analysis, PCR-based and next-generation sequencing (NGS)-based approaches are explored. While PCR remains the backbone for ctDNA analysis, this approach is limited to CRC-specific driver mutations or requires prior knowledge of genetic alterations associated with the investigated CRC tumor. However, PCR-derived techniques are cost-affordable compared with NGS and allow the identification of therapeutically actionable targets or acquired mutations responsible for resistance to therapy. By qPCR-derived techniques, therapeutic vulnerabilities of CRC patients can be easily identified, allowing to discriminate between patients that are suitable for a particular treatment regimen or to readjust treatment according to acquired mutations. Currently, for mCRC patients, fluoropyrimidines remain the backbone of more intensive treatment strategies, including doublet (FOLFOX or FOLFIRI) or triplet combinations (FOLFOXIRI) with leucovorin, oxaliplatin (OXP), irinotecan (IRI), and capecitabine (CAPE) (143–145), added to target agents such as bevacizumab and/or anti-EGFR (146). With this respect, pivotal phase 3 randomized studyAVF2107g proves that the addition of bevacizumab to FOLFIRI significantly improved the overall survival. More, based on promising results obtained in phase 2 studies on the combination of bevacizumab with a FOLFOXIRI, a phase 3 randomized study (TRIBE) showed that this approach displays improved progression-free survival among patients with mCRC as compared with FOLFIRI and bevacizumab (hazard ratio for progression, 0.75; 95% CI, 0.62 to 0.90; P = 0.003) and and reached a median OS of 37.1 months in the RAS wild-type (147). More, the addition of cetuximab (CET) and panitumumab (PAN), which are anti-EGFR drugs, to FOLFOX or FOLFIRI chemotherapy in mCRC patients with KRAS exon 2 wild type and overexpression of EGFR led to improved results with respect to objective response rate, progression-free survival and OS (148). The main concern in the association of anti-EGFR drugs with triplet therapy regimens is the significant toxicity rate in therms of diarrhea, asthenia, mucositis and neutropenia. However, previous analysis of some pharmacogenomics markers such as 5-FUDR for 5-FU degradation rate or and single nucleotide polymorphisms (SNPs) in ABCB1, CYP3A4, DYPD, UGT1A1 which encode for enzymes responsible for 5-FU metabolism, are usefull to predict chemotherapy induced gastrointestinal toxicity (148). Consequently, a shift towards the administration of biological therapies is observed based on the success of these to improve the OS of mCRC patients, among which the best outcomes were observed following treatment with human vascular endothelial growth factor (VEGF) monoclonal antibody bevacizumab and the EGFR monoclonal antibodies cetuximab and panitumumab (149, 150). Independent of the therapeutic choice, the success of the therapy is based on the correct identification of the existing patients’ genetic abnormalities. In contrast to tissue biopsy, identifying genetic alteration by ctDNAs analysis can be employed on-demand establishing, therefore, a patient’s real-time treatment sensitivity pattern that helps avoid the administration of drugs that lack their antitumor effects and exert unnecessary toxicity. More, in absence of tumor tissue for CRC patient molecular characterization, blood can be used for ctDNA mutation screening, therefore providing a correct and efficient targeted therapy scheme (151).

Several genes alterations are screened for aiding CRC treatment, most usual for identifying anti-EGFR-resistant-CRC (152). EGFR is a transmembrane receptor tyrosine kinase with a crucial role in CRC development and progression, being, therefore, an important target for therapy by administration of EGFR monoclonal antibodies or tyrosine kinase inhibitors (153). Following administration of EGFR monoclonal antibodies, these bind to the extracellular domain of EGFR and prevent the activation of the receptor tyrosine kinase and multiple downstream signaling pathways related to cell survival, proliferation, and migration, such as RAS-RAF-MAPK, PI3K-PTEN-AKT, and JAK/STAT pathways (154). Half of the CRC patients harbor RAS mutation, a particularity that renders the anti-EGFR drugs ineffective (155). While KRAS-mutated patients are not exclusively associated with EGFR resistance, mutations in KRAS codons 12 and 13 at exon 2 are clear indicators of EGFR therapy resistance (156, 157), together with mutations in exons 3 and 4 that can indicate a poor response to anti-EGFR (149, 158–160). Another mutation that can overdrive the MAPK pathway, thus promoting cell survival and proliferation, is BRAF identified in 5% - 10% of CRC cases, the commonest being the BRAF^V600E^ mutation (>95%) (161). The presence of BRAF mutation is an indicator of poor prognosis in patients with mCRC and a marker of resistance to anti-EGFR therapy, and it was considered to be a signature of patients not harboring RAS mutations (154, 162, 163). However, recent studies showed the presence of concomitant KRAS and BRAF mutations (164–167). Other EGFR-resistant related mutations are identified with significantly low frequency than KRAS and BRAF mutation, making it difficult to unravel their role in anti-EGFR therapy resistance. The phosphatidylinositol-4,5-bisphosphate 3-kinase (PI3K)-Phosphatase and Tensin Homolog (PTEN) pathway is an oncogenic signaling network that is deregulated in CRC by aberrant activation of the prooncogenic gene PI3K or through loss of function of the tumor suppressor PTEN (43). PI3K mutations are identified in 10-20% mCRC in exon 9 or exon 20 and lead to constitutive activation of the p110a protein kinase and its downstream pathway, which promotes tumor cell proliferation and survival (168, 169). Both PI3K mutations and loss of PTEN function have been associated with EGFR blockade resistance (170, 171). More, although HER2 amplification was considered relevant for breast and gastric cancer, it appears that it represents also a negative predictor for anti-EGFR therapy (172). With a lower rate of incidence (2% - 4%), MET amplification can determine resistance to anti-EGFR drugs (173).

KIT proto-oncogene receptor tyrosine kinase and platelet-derived growth factor receptor (PDGFR) mutations play a key role in the prognosis of gastrointestinal stromal tumors (4), while ALK receptor tyrosine kinase fusion genes (174) and beta-catenin 1 (CTNNB1) mutations (175, 176) are crucial in inflammatory myofibroblastic tumors and desmoid fibromatosis, respectively. More, the key feature of the CRC occurring neuroendocrine neoplasms (NENs) classification is the discrimination between well-differentiated neuroendocrine tumors (NETs), the carcinoid tumors, and the poorly differentiated neuroendocrine carcinomas (NECs). Despite that NETs and NECs share the expression of neuroendocrine markers, they display significant genetic differences, and therefore, they are not considered to be akin neoplasms (177–180). The main genetic differences between them rely on the Retinoblastoma (RB) and Tumor Protein p53 (TP53) mutations that are commonly displayed by NECs. High-grade colorectal NECs are one of the most lethal types of neuroendocrine neoplasm (181). This poor prognosis pushes the exploration of more effective treatments. Late studies aimed to unreveal colorectal NECs genomic fingerprint. Considering the low incidence of colorectal NECs, only a few studies included highly significant amounts of patients. However, some noteworthy findings show that BRAF gene is frequently altered (182–186). Chen et al. (187) explored the genomic characteristics, and potentially targetable gene alterations in colorectal NEC and compared these characteristics with those of colorectal adenocarcinomas and gastrointestinal NETs using the American Association of Cancer Research (AACR) Project Genomics, Evidence, Neoplasia, Information, Exchange (GENIE) public NGS database (188). They discovered that the genetic characteristics of colorectal NEC are more similar to colorectal adenocarcinomas than gastrointestinal NETs and that the Wnt, MAPK, and PI3K signaling pathways as well as the cell cycle regulation are frequently aberrant in colorectal NECs. More, they confirmed that a significant group of colorectal NEC patients was bearing potentially targetable alterations such as BRAF^V600E^ (187).

There is no doubt that the identification of actionable mutations in mCRC has significantly contributed to the expansion of the therapeutic options available for these patients, but unfortunately very few of the drugs used are at the moment FDA-approved. However, besides the use of monoclonal antibodies to target EGFR, anti-angiogenic and anti-kinase drugs are also used for mCRC management, and studies have shown that these can significantly increase OS and PFS of mCRC patients. Generally, anti-angiogenic and anti-kinase agents are combinatorically administrated with fluoropyrimidine-based chemotherapy plus OXP and/or IRI (189, 190). Among these, the following are clinically approved and used in mCRC treatment regimens: bevacizumab (VEGF inhibitor), aflibercept (blocks activation of VEGF-A, VEGF-B, and PIGF), ramucirumab (anti-VEGFR-2 monoclonal antibody), and regorafenib (191) with approval and reimburse differently applied in the countries. This last one is a multikinase inhibitor that not only acts on angiogenic protein kinases (VEGFR-1, VEGFR-2, VEGFR-3, TIE-2), but as well on proteins involved in oncogenesis (KIT, RET, RAF-1, BRAF, BRAF^V600^), metastasis (PDGFR, FGFR), and tumor immunity (CSF1R) (192, 193). Therefore, as in CRC cases mutations in genes encoding protein kinases are frequently identified, these drugs can be used to target and block the activity of these overexpressed proteins, improving, therefore, the OS and PFS of mCRC patients (194).

Based on the low content of ctDNA, its small fragment size, and limited high-life, qPCR-based methods were insufficient to detect mutations in ctDNA due to their limited sensitivity. Therefore, new techniques such as droplet digital PCR (ddPCR) or its high-throughput version BEAMing (beads, emulsion, amplification, and magnetics) showed superior efficiency in ctDNA analysis. The main advantage of ddPCR is the use of nanoliter-sized water-in-oil emulsion droplet technology to partition the cfDNA sample into numerous independent PCR sub-reaction (195), the target sequence being therefore concentrated between the aqueous droplets. In this way, after detection by fluorescence of the amplified targeted sequences and comparison of the signals obtained, rare mutations are discriminated from the wild-type sequences. ddPCR principle by which multiple PCR individual microreactors are generated, favors the quantification of low-abundance point mutations in cfDNA from a background of wild-type sequences, with high sensitivity ranging from 0.001% to 0.1% (173, 196).

In contrast, the modern NGS platform opens new opportunities for CRC patients by employing massively parallel sequencing techniques for genome-wide assessment and screen for multiple mutations with greater sensitivity without prior knowledge of the patient`s existing genetic abnormalities and can lead to the discovery of novel therapeutic targets (197). Undoubtedly, the main advantage of NGS analysis compared with the targeted approaches is the possibility of investigating a large range of markers reaching up to several hundred genes in a single panel. Also, a single test provides valuable information on single nucleotide polymorphisms, short indels, copy number variations, gene fusions, TMB (tumor mutational burden), or MSI (198). New NGS panels have been developed to detect the pathological variants associated with cancer at both cfDNA and cfRNA levels (e.g., Oncomine Pan-Cancer Cell-Free Assay - Thermo Fisher, Waltham, MA, USA). NGS approaches are based in fact on massively parallel sequencing technologies that offer high throughput, reproducibility, and speed. High-throughput sequencing, which includes Next-Generation “short-read” and “long-read” Sequencing methods allows scientists to perform a wide variety of applications in terms of genomics such as genome and exome *de novo* sequencing and resequencing, study of DNA-protein interactions, and epigenome characterization. Nowadays, there are at least 9 different NGS platforms using different template preparation and sequencing chemistry. However, all NGS platforms perform sequencing of millions of small fragments of DNA in parallel producing thousands or millions of sequences concurrently. Bioinformatics analyses are used to piece together these fragments by mapping the individual reads to a reference. NGS platforms allow deeply sequence target regions, analyze epigenetic factors such as genome-wide DNA methylation and DNA-protein interactions, and also sequencing cancer samples to study rare somatic variants or tumor subclones. Many methods are applying NGS to target panels for specific and highly sensitive detection of targeted ctDNA mutations (Tagged-Amplicon deep sequencing - TAm-seq; Safe-Sequencing System - Safe-SeqS or CAncer Personalized Profiling by deep sequencing - CAPP-Seq) (199). However, the use of NGS for detecting rare mutations provides a lower technical sensitivity than ddPCR (200), highlighting that PCR-based methods and NGS could be employed as complementary techniques for clinical applications.

Various commercial platforms are available for identifying actionable mutations in CRC such as Idylla or OncoBEAM, Idylla platform being used also for MSI detection (201–203). Vidal and colleagues (204) revealed a 93% overall agreement between the mutational status of RAS when comparing tissue and plasma samples. From 55 patients presenting RAS mutations in investigated tumor tissue, 53 had RAS mutational status confirmed by ctDNA analysis using OncoBEAM. Poor results were obtained by García-Foncillas et al. that showed just 89% agreement between results obtained by analyzing the mutational status of KRAS from tissue vs. plasma (201). For detecting MRD, Tie et al. (205) successfully used massively parallel sequencing-based assays to predict recurrence in patients with resected stage II CRC by analyzing ctDNA from postoperatively blood samples and can measure in real-time the effectiveness of treatment for patients receiving adjuvant chemotherapy.

Other valuable biomarkers in the liquid biopsy are ctRNAs such as micro-RNA (miRNA), messenger RNA (mRNA), and noncoding RNA (ncRNA) that are usually released in circulation within extracellular vesicles or associated with RNA-binding proteins (206). ncRNAs for example, are involved in all CRC stages of tumorigenesis and progression influencing key signaling pathways such as: WNT/-catenin, phosphoinositide-3-kinase (PI3K)/protein kinase B (Akt), epidermal growth factor receptor (EGFR), NOTCH1, mechanistic target of rapamycin (mTOR) and TP53 (207). Although an incomplete domain, in the last years many gene fusions have been identified in colorectal cancer only due to the possibility of high throughput NGS. The most frequent gene rearrangements detected in CRC with prognostic value and treatment perspectives are: NTRK fusions, ALK and ROS1 rearrangements, RET fusions and BRAF translocations. NTRK fusions were detected especially in women, elder people with RAS and BRAF wild-type genes and were presumably associated with primary resistance to EGFR-targeted treatment. Conventionally gene fusions are investigated by FISH, IHC or RT-PCR. Unfortunately, these methods can test for only one fusion gene at a time, being low-throughput and sometimes costly. The great advantage NGS brings in clinics is the large amount of information obtained. In just one test hundreds of gene rearrangements, known and unknown, frequent or rare can be detected. It can also investigate very small rearrangements that would be impossible to be evaluated using classical methods (208).

## 4 Biomedical Applications of Artificial Intelligence in Colorectal Cancer Management

### 4.1 General Background

Nowadays we are all surrounded and widely interacting in our daily lives with AI in the form of consumer technology, whether we think about smartphones, wearable devices, search engines, social media channels, personalized advertising, facial recognition, autonomous vehicles, energy-efficient buildings, smart toys and many more. The concept of AI is not new (209), but only in the past few years, it has raised interest due to its potential as a virtual assistant able to serve various domains of human activity, especially the biomedical field. These AI experiences aim to improve human lives by increasing efficiency and by tailoring solutions for each individual. From this point of view, AI may be defined as a combination of theories, algorithms, and computing frameworks enabling a machine to independently reproduce intellectual processes associated with human cognition to decide on an action in response to its perceived environment to achieve a predetermined goal. Consequently, AI empowers humans to become faster in analyzing big amounts of data and smarter in decision-making by augmenting, however without replacement, human intelligence, and intuition (210). At its core, AI is a branch of computer science that has gradually changed not only our daily lives but also the landscape of healthcare and biomedical research for medical image analysis, intraoperative imaging, and genomics.

Starting with the ‘50 when the concept was first defined, there have been developed rule-based clinical decision support systems (211) that helped physicians to diagnose disease (212, 213) or decide the best treatment (214). Despite all these advantages and clear progress in the current practice, the rule-based systems displayed a series of inconveniences such as high building cost and system limitation by the difficult encoding of complex interactions and requirement of solid medical knowledge (215). Therefore, this first generation of AI systems has been improved by the integration of ***machine learning (ML) methods***, which apply complex interactions to identify data patterns. ML is a subfield of AI that uses both mathematical and statistical approaches to improve the performance of computers. More specifically, ML resides in the development and implementation of dynamic algorithms that are able to take particular actions in response to particular inputs (environment), analyze the data to determine the actions (216, 217). These algorithms are able to self-improve (learn) as more data is available and the optimization process is called “training”. Depending on the tasks to be solved, basic ML algorithms can be divided into supervised, unsupervised (218), and reinforcement learning.

***Supervised learning (SL) algorithms*** such as Naive Bayes classification (217), linear and logistic regression (219), support vector machines (SVMs) (216), or random forests (RF) (220) analyses the training data (input-output pairs) and map an input to output (221). A good example, in this case, is the histopathology grading of digitalized tissue biopsy slides. First, a set of images are labeled by the pathologists as positive or negative for a specific type of cancer and then SVMs for example learn to classify new, unlabeled images and tag them on a “probability map”. Zhi et al. (222) used five data sets containing metastatic and non-metastatic CRC samples to identify potential biomarkers for CRC metastasis. For this, the authors used a meta-analysis method to identify the metastatic and non-metastatic CRC differentially expressed genes and then, used the SVM classifier to select the top candidates having as reference a CRC dataset from The Cancer Genome Atlas database. The authors report a 100% precision of the SVM-classified 40 gene signatures and highlight the CREB1, CUL7, and SSR3 genes as biomarkers for the prognosis of metastatic CRC.

***Unsupervised learning (UL) algorithms*** such as k-means clustering (217), principal component analysis (223), and autoencoders (224) identify patterns from untagged data by separating the items into different classes based on the training data features to find sub-clusters and outliers in the data. Bae et al. (225) proposed a feature selection method able to distinguish CRC patients from normal individuals using K-means clustering and the modified harmony search algorithm. To classify CRC using gene information the authors analyzed 6500 genes in 40 CRC tissue biopsies and 22 normal colonic tissue samples by a 4 step hybrid method consisting of: (i) Z-normalization of gene information values, (ii) Fisher score based reduction of redundant genes, (iii) K-means clustering of representative genes and (iv) Harmony Search (HS) algorithm based selection.

***Reinforcement learning (RL)***, consists of a set of algorithms (agents) that operate sequentially by predicting the features of each step based on the past and present features while assigning a reward or a penalty on the prediction basis. The purpose of RL is for the agent to learn an optimal or nearly-optimal policy that maximizes the “reward function” (226).

***Deep learning (DL)*** is a subfield of ML which involves training an artificial neural network (ANN) with many layers. DL recapitulates the biological neural network of the human brain and uses a layered structure of algorithms to analyze data, identify patterns, draw conclusions, and make decisions. The basic architecture of deep neural networks (DNNs) consists of an input and an output layer together with a variable number of hidden layers in between. In such a network, the input is provided to the input layer, which transfers its computed value towards the hidden layers that are finally linked to an output layer. DL can be further classified into the deep neural network (DNN), recurrent neural network (RNN), and convolutional neural network (CNN). CNNs are particularly important to identify patterns from unprocessed images (227). CNNs apply nonlinear transformations to structured data, for example the raw pixels within an image, to automatically learn relevant features. In this approach, it is mandatory to process accurately the images before analysis in order to reduce the risk of the model to learn from artifacts. CNNs basically uses two main models: the first one uses images from a large collection such as ImageNet to train the initial layers, and the second one is based on an auto encoder where the model learns background features from a subset of representative images (228). DL has been widely applied to empower medical procedures mainly involving analysis of images resulting from a wide range of procedures (229–232) and genomics (233).

**Figure 2:**
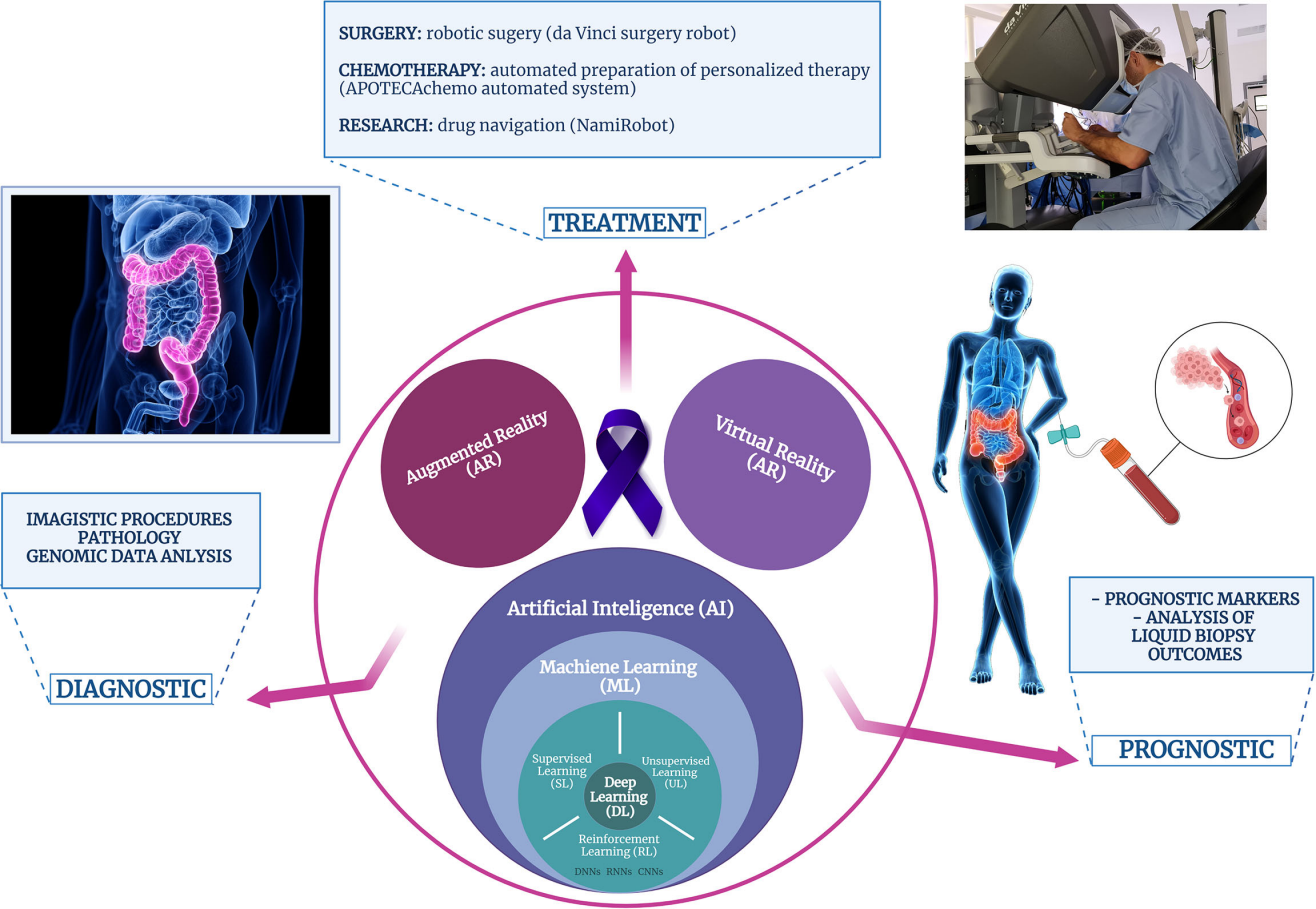
Schematic representation of the computer-assisted technologies applications in the biomedical field, particularly, in patients with colorectal cancer.

In brief, biomedical applications of AI can be divided into two main branches: virtual and physical. The virtual component consists of ML algorithms: supervised learning, unsupervised learning, and reinforcement learning, and DL as an ML subset (**Figure 2**). CNN is the most prominent DL construction, representing a particular type of multilayer artificial neural network that is highly efficient for image classification (227). While the virtual branch of AI-powered biomedical applications refers to image analysis and genomic (big data) analysis, the physical component includes medical devices, surgery robots, and devices for automated therapy formulation and nanorobots for targeted drug delivery (234).

***Virtual reality (VR)*** is a simulated experience that generates an immersive, artificial image and/or environment with real-time interaction. Applications of VR include entertainment (video games), education (virtual training in a wide range of fields including medicine), and even business (virtual meetings). In medicine, one of the earliest VR platforms developed was Minimally Invasive Surgical Trainer-Virtual Reality (MIST-VR) (Mentice Medical Simulation, Gothenburg, Sweden) for endoscopic training (235). VR 3D technology is being used for simulation-based training due to its major advantage of creating near true experiences overcoming the disadvantages of the current distance learning and 2D videos (236). Currently, using VR (237), previously segmented organs and structures can be highlighted by streaming a digital full image, often visualized as a 3D render in a virtual environment.

***Augmented reality (AR)*** is an interactive experience consisting of the addition of artificial information to the objects in the real world through computer-generated perceptual information that enables the user to perform tasks easily. This can be achieved across multiple sensory modalities like overlapping images and generating video or computer models. The first headset equipped with an overlapping display was introduced by Ivan Sutherland in 1965 and served for military purposes (238). Some examples with applications in the biomedical field are AccuVein (AccuVein Inc., NY, USA), a projector-like device that displays a map of the vasculature on the skin surface (239), or Google Glass which is a head-mounted display with generated objects superimposed onto real-time images. Unlike the virtual environment provided with VR, AR has been used in real surgery for many years (240) as it can overlay “invisible” information such as pre-or intraoperatively obtained imaging findings in the real environment.

### 4.2 AI in CRC Screening and Diagnostic

CRC is a highly preventable disease with very good survival rates when diagnosed in the early stages. This is why routine screening is a crucial step in lowering the incidence rates of this pathology (241) while offering patients the hope for disease-free. The premalignant alterations from normal to malignant lesions take almost over 15 years. Efficient screening methods are permanently developed to accurately diagnose abnormal modifications with potential pathologic signification (242, 243). Available CRC screening procedures include colonoscopy, capsule endoscopy, imaging examinations, and/or blood and stool tests (244, 245). ML algorithms may be used as non-invasive and cost-effective methods to screen the CRC risk in large populations using personal health data (246).

#### 4.2.1 AI in CRC Imagistic Screening Procedures

##### 4.2.1.1 Colonoscopy and Virtual Colonoscopy

Colonoscopy is currently the gold-standard method for CRC screening displaying several advantages such as high sensitivity, specificity, and ability to directly visualize the tissue and act on cancerous and precancerous lesions by polyp resection and/or tissue biopsy harvest. Multiple works have shown that colonoscopy significantly reduces CRC incidence and mortality (247–250). However, due to the significant risk of the human eye to missing spotting small or flat polyps, computer-aided detection (CADe) and diagnosis (CADx) systems were developed to assist colonoscopy in real-time automated polyp detection (251). DL techniques such as CNNs algorithms were used to improve colorectal adenoma detection rates and proved to accurately spot the presence of premalignant lesions (252). With this respect, the DL-based CADe model effect on polyp and adenoma detection rates was investigated by Wang et al. (253) in a randomized controlled trial, while the CADx potential assistance in discrimination between neoplastic and non-neoplastic polyps during a colonoscopy was investigated by Mori et al. (254). To improve even more the CRC polyp detection and classification, AI-based tools were employed furthermore in colonoscopy. This approach led to the development of the virtual colonoscopy or computed tomographic colonoscopy described for the first time by Lefere in 2006 (255) and originating from the computed tomographic colonography described in 1994 (256, 257). This AI-powered tool may also contribute to the automatic detection of flat neoplastic lesions, which may represent an aggressive tumorigenesis and a determining factor in increased adenoma miss rate (258). Numerous strategies for small and/or flat polyps detection and their discrimination between neoplastic and non-neoplastic were developed and already reviewed in recent literature (259).

##### 4.2.1.2 Capsule Endoscopy (CE)

Capsule endoscopy (CE) is a minimally invasive diagnostic technique, well tolerated by patients, and a powerful alternative approach in incomplete colonoscopy cases. Traditionally colon CE is a time-consuming procedure requiring human interpretation and analysis of the captured images for the detection of potential CRC lesions. In this view, AI-powered technologies have the potential to increase the detection rates of adenomas by automating the analysis of the results (260, 261) reported two algorithms, one able to match CE with polyps identified by colonoscopy and another one based on deep CNNs for automatic colorectal polyp detection and localization.

#### 4.2.2 AI in CRC Molecular Imaging

Molecular imaging is a modern approach of medical imaging that consists of the *in vivo* visualization of anatomical structures based on the presence of a radioactive, fluorescent, or magnetic label. This approach can be used for early diagnostics, therapy planning, and resection. Nuclear medicine remains the gold standard for non-invasive molecular imaging, using PET imaging, combined with CT or MRI anatomical imaging (262). Alternatively, an optical or electrical expression of the target molecule in a non-contrasted fashion (263, 264) is applied. More, the concept of interventional molecular imaging refers to the use of tracers and other surgical molecular imaging approaches, to target and help surgeons precisely resect tissues based on molecular features (265) during radio-guided surgery (266, 267) or fluorescence-guided surgery (268).

##### 4.2.2.1 AI in CRC Molecular Imaging for Diagnostic

Molecular imaging powered by AI strategies holds great potential to improve the CRC diagnosis as AI algorithms allow the comparison of the information available for a current patient with huge databases of previously treated patients potentially empowering the selection of the most optimal treatment strategy and the prediction of the treatment outcome (269).

***Magnifying chromoendoscopy*** is an optical diagnosis procedure, that proved to spot colorectal lesions with over 90% of sensitivity, specificity, and accuracy (270). This technique consists in the analysis of the polyp surface with a high-resolution magnifying colonoscope after indigo carmine or crystal violet dye spray. With this respect, Häfner et al. (271) developed a texture feature extraction algorithm, while Takemura et al. (272) used the quantitative analysis of pit patterns to propose a software model for the differential diagnosis of colorectal lesions.

***Endocytoscopy*** refers to an endoscopic imaging procedure, which is an *in vivo* imaging microscopy technique that allows a real-time diagnosis based on the analysis of the cells patterns observed at high magnification (273). Endocytoscopy relies on the contact light microscopy principle and requires colonic mucosa prestaining with absorptive contrast agents, such as toluidine blue (273, 274). Computer-assisted algorithms were developed for *in vivo* discrimination of colonic lesions using endocytoscopy and even further improved by the use of SVM as classifiers for benign, adenomatous lesions or invasive carcinoma (275).

***Confocal laser endomicroscopy*** is a microscopic imaging technique powering the *in vivo* inspection of cellular and subcellular structures (276). K-nearest neighbor classification was used by Andre et al. (277) to build an automated polyp characterization system that proved to distinguish between malignant and benign lesions with an accuracy rate of almost 90%.

***Laser-induced fluorescence spectroscopy (LIFS) and autofluorescence imaging (AFI) endoscopy*** are used for *in vivo* detection of targeted tissue fluorescence emission. The autofluorescence could emerge from endogenous molecules such as collagen or flavins after excitation with an appropriate light source. Based on color differences these procedures can predict in real-time the lesion pathology and accurately characterize the colorectal polyps. Using computer-aided software for color analysis models these techniques may significantly contribute to the differentiation of non-neoplastic from neoplastic lesions during colonoscopy (278–281).

##### 4.2.2.2 CRC Surgery Procedures Powered by Molecular Imaging and AI

Classically, preoperative imaging techniques are routinely used to create detailed roadmaps that help surgeons to plan the surgery and execute the desired resection. However, the up growing interest in the development of minimally invasive surgical strategies, such as robotic surgery has empowered molecular imaging as a valuable tool for precision surgery. Intuitive AR visualizations and AI integration for an optimized perception into the video console used by surgeons in robotic surgery brought the classical practice to the next level. For example, the TilePro extension of the da Vinci surgical console shows preoperative images as windows in the surgeon’s display (282, 283). Beyond planning and guidance through the preoperative scans, intraoperative molecular imaging approaches (radioactive, fluorescent, magnetic, multispectral optoacoustic tomography (284), fiber-based microscopy (285), Raman spectrometry (286), etc.) play an important role in intraoperative lesion localization, decision making, and subsequent confirmation.

#### 4.2.3 AI in CRC Imagistic Pathology Diagnostic

Despite that other tools have been proposed with great promise for the future, tissue biopsy remains the gold standard for colon cancer diagnosis and staging. Therefore, much effort has been invested to improve this approach. With this respect, some groups have developed support-vector machines (SVMs) to enable the automatic classification and diagnose CRC based on biopsy samples, significantly improving the accuracy of diagnosis while reducing time and costs (287–289). Convolutional neural networks (CNNs) are the most commonly used AI technology in pathology image analysis (290). For example, CNNs were used to detect and classify nuclei in colon cancer biopsies (291). Histopathology images of cancerous tissues stained with standard hematoxylin and eosin (HE) stains were used to develop technologies with significantly enhanced reading accuracy (291). More, the TuPaQ algorithm using immunohistochemistry (IHC) staining was developed to segment CRC tumor epitheliums, providing a basis for automated biomarker quantification (292). Recently, Yu et al. (293) developed a recognition system for CRC which achieved one of the highest diagnosis accuracies in cancer diagnosis with AI using SL. More, AI has made a huge step in the field of intraoperative pathology, providing preliminary evaluations or highlighting suspicious areas (294). For example, one-step nucleic acid amplification (OSNA), a non-imaging approach of the intraoperative biochemical analyses of lymph nodes has been successfully implemented into the clinical routine in several countries showing excellent diagnostic accuracy in CRC (295).

#### 4.2.4 AI in CRC Genomic Data Analysis

AI algorithms have shown several promising results with genetic testing for CRC. For example, Hu et al. (296) classified CRC patients with the Union for International Cancer Control (UICC) II into two groups based on their relapse status and compared the classification accuracy using the following neural networks: S-Kohonen (91%), Back-propagation (BP, 66%), and SVM (70%). Xu et al. (297) used SVM to identify differentially expressed genes and identified a 15-genes with relevance in distinguishing patients with a high CRC recurrence risk. Zhang et al. (298) developed a counter propagation artificial neural network (CP-ANN) able to detect BRAF mutation. AI algorithms such as but not limited to BP were used by Wang et al. (299) to combine gene expression profiling data from The Cancer Genome Atlas (TCGA) database for CRC diagnosis improvement. In the context of the up growing interest of the liquid biopsy approach development, Wan et al. (300) proposed a ML method using tumor-derived cfDNA. Chang et al. (301) compared the expression profiles of 380 miRNAs in stage II CRC versus normal tissue and identified a 3-miRNA signature potentially predicting the tumor status in stage II CRC. However, during this time, many AI-based technologies were developed targeting the using miRNAs (302–305). Ge et al. (306) used CIBERSORT, a deconvolution algorithm, to study the role of 22 immune cells types and 404 immune-related gene expression in the CRC surrounding microenvironment.

Liquid biopsies output data are large and complex and therefore, traditional methods fail their efficient process. In this view, ML represents a promising tool for automated analyses of these data and future prediction (307). Different ML algorithms, such as SVM, random forest (RF) and ANNs, have been widely used in the field of medicine (308–310). SML is usually implemented for liquid biopsy data analysis and involves model evaluation and selection methods. Data preprocessing consists of missing-value solution, normalization, dimension reduction, and feature reconstruction. Regarding the CTCs in particular, the CellMax (CMx^®^) platform was developed based on AI achieved clinical sensitivity and specificity of 80% (121). In addition, ML algorithms could assist in analyzing data on specific serum protein biomarkers such as LRG1, EGFR, ITIH4, HPX and SOD3 in order to identify CRC with 70% sensitivity at over 89% specificity (311).

### 4.3 AI in CRC Surgery

Along with chemotherapy administration, surgery remains the main curative procedure for CRC management. With the development of robotic surgery as a minimally invasive procedure, CRC management enters a new era. To date, the da Vinci System developed based on the physical branch of AI technologies, is the most used surgical robot worldwide. The major advantages of its use reside both on the surgeon’s and patient’s sites. Robot-assisted surgery needs smaller incisions that will conduct minimal scarring and a significant decrease in the risk of surgical site infections, faster recovery and decrease of hospitalization period. Postoperative pain and bleeding are significantly lower as compared to traditional open surgery (312, 313). On the other side, using computer-controlled devices surgeons benefit from enhanced visual field, more flexibility, increased precision, and minimal fatigue. Besides the above-mentioned advantages, the da Vinci dual-console also allows integrated teaching and supervising. Several retrospective studies on robotic-assisted CRC resections showed that the procedure significantly reduced the complications rate and resulted in less evident inflammatory response as compared with the open surgery (314, 315). Other robots for surgery such as The Senhance System (TransEnterix Surgical Inc., Morrisville, NC, USA) which is a laparoscopy-based system are developed. Overall, robot-assisted colorectal surgery has better performance in terms of both short- and long-term outcomes (316).

### 4.4 AI in CRC Therapy

Concerning the actual preparation of the administered therapy for patients, manual handling of cytotoxic drugs is a high-risk activity due to the prolonged exposure to carcinogens (317) and also a major error generating point in the patient’s management (318). Therefore, the introduction of robots such as the APOTECAchemo automated system (319) improved the safety of cytotoxic drug preparation, almost excluding the errors associated with human handling (320). Regarding CRC therapy research, Martel et al. (321) developed the NamiRobot System that can deliver drugs to cancer cells and hypoxic regions based on the reduced oxygen levels caused by the proliferation of cancer cells. More, AI technology can also promote research on new drugs. For example, Cruz et al. (322) used machine learning to detect with a prediction accuracy of over 63% the half-maximal inhibitory concentration (IC50) of a new drug on the HCT116 colon cancer cell line.

## 5 Conclusions and Perspectives

The emerging field of research in CRC pathology empowered the development of new approaches and technologies for better management of this malignancy in terms of: diagnostic, treatment, and prognostic. Specifically, on one hand, liquid biopsy is a promising tool revealing the residual cancer cells, and patient’s disease progression in real-time. This valuable outcome is enabled by the access to worthy decision-making information like genomic profiling for eligibility to targeted therapy, identification of chemotherapy-induced genomic alterations as compared to the initial tissue biopsy, detection of disease relapse, and/or MDR. Moreover, this innovative approach could be applied in clinical practice to enhance the knowledge of the specific biological feature of CRC disease including temporal and spatial heterogeneity. For example, the analysis of NGS data might reveal RAS mutations, which predict resistance to epidermal growth factor receptor (EGFR) therapy and/or BRAF mutations which indicate that encorafenib and cetuximab therapy might be effective. Moreover, liquid biopsy is a powerful tool also for driving anti-EGFR rechallenge therapy in mCRC as shown within CHRONOS, a phase 2 trial aiming to rechallenge anti-EGFR therapy by monitoring of the mutational status of RAS, BRAF and EGFR in circulating tumor DNA (ctDNA) (323). Lastly, but not least, the same analysis might spot NTRK fusions or the amplification of ERBB2 which can be associated with dual anti-HER-2 blockade. On the other hand, the development of AI-based technologies for biomedical applications opened a new era in the field of automated detection of colorectal polyps during screening procedures such as but not limited to colonoscopy and automated detection of malignant tissues in pathology analysis. More, AI meets AR and VR in robotics, with specific applications in CRC surgery (example: the highly versatile da Vinci robotic surgery system) and also in traceable and personalized proper preparation of anti-blastic drugs in hospitals (example: APOTECAchemo automated system).

## Author Contributions

OG reviewed the whole content regarding CRC pathology, screening methods and robotic surgery. He also contributed with content for the images. AH wrote and reviewed the whole section 3. MZ, AS, MM, and IB wrote sections 4.1, 4.2 and 4.3. LB, SG, and MC wrote and reviewed the content regarding ctDNA (Section 3.2.). CNe wrote section 4.4. CNi wrote section 2 and BG wrote sections 1 and 5, reviewed the whole manuscript and coordinated the whole team. All authors contributed to the article and approved the submitted version.

## Funding

This work was supported by a grant of the Romanian Ministry of Education and Research, CCCDI - UEFISCDI,project number PN-III-P2-2.1-PTE-2019-0577, within PNCDI III.

## Conflict of Interest

Author LB was employed by company OncoTeam Diagnostic S.A.

The remaining authors declare that the research was conducted in the absence of any commercial or financial relationships that could be construed as a potential conflict of interest.

## Publisher’s Note

All claims expressed in this article are solely those of the authors and do not necessarily represent those of their affiliated organizations, or those of the publisher, the editors and the reviewers. Any product that may be evaluated in this article, or claim that may be made by its manufacturer, is not guaranteed or endorsed by the publisher.
